# Accelerating String Comparison in RLZ Compressed Sequences via LCE Jumps

**DOI:** 10.64898/2026.06.11.731742

**Published:** 2026-08-10

**Authors:** Rahul Varki, Christina Boucher

## Abstract

Relative Lempel–Ziv (RLZ) is an effective compression method for large, repetitive collections; however, the fundamental primitives required to elevate it from a passive archival format to a tractable representation for compressed construction have yet to be fully established. In this paper, we introduce an algorithmic framework for structurally comparing and lexicographically sorting sequences of RLZ factors. We characterize when direct factor comparisons are necessary and when they can be bypassed using RLZ specific shortcuts. We further introduce a method for extending truncated factors into right-maximal matches, enabling the recovery of matching statistics from the RLZ parse. Experimentally, RLZ sorting achieved speedups of up to 3.93× over character-based sorting. Together, these results advance the use of the RLZ format as a foundation for compressed construction.

## Full Text Availability

The license terms selected by the author(s) for this preprint version do not permit archiving in PMC. The full text is available from the preprint server.

